# Predictive biomarkers for death and rehospitalization in comorbid frail elderly heart failure patients

**DOI:** 10.1186/s12877-018-0807-2

**Published:** 2018-05-09

**Authors:** Cristina Pacho, Mar Domingo, Raquel Núñez, Josep Lupón, Julio Núñez, Jaume Barallat, Pedro Moliner, Marta de Antonio, Javier Santesmases, Germán Cediel, Santiago Roura, M. Cruz Pastor, Jordi Tor, Antoni Bayes-Genis

**Affiliations:** 1 Servei de Medicina Interna i Unitat de Geriatria d’Aguts, Hospital Universitari Germans Trias i Pujol, Badalona, Barcelona, Spain; 2 Servei de Cardiologia i Unitat d’Insuficiència Cardíaca, Hospital Universitari Germans Trias i Pujol, Badalona, Barcelona, Spain; 3 Servei de Bioquímica i Anàlisis clíniques, Hospital Universitari Germans Trias i Pujol, Badalona, Barcelona, Spain; 4 Department de Medicina, Universitat Autònoma de Barcelona, Barcelona, Spain; 5 CIBERCV, Instituto de Salud Carlos III, Madrid, Spain; 6 Cardiology Department, Hospital Clínico Universitario, INCLIVA Valencia, Valencia, Spain; 7 Departamento de Medicina, Universidad de Valencia, Valencia, Spain; 8 ICREC Research Program, Germans Trias i Pujol Health Science Research Institute, Badalona, Spain

## Abstract

**Background:**

Heart failure (HF) is associated with a high rate of readmissions within 30 days post-discharge and in the following year, especially in frail elderly patients. Biomarker data are scarce in this high-risk population. This study assessed the value of early post-discharge circulating levels of ST2, NT-proBNP, CA125, and hs-TnI for predicting 30-day and 1-year outcomes in comorbid frail elderly patients with HF with mainly preserved ejection fraction (HFpEF).

**Methods:**

Blood samples were obtained at the first visit shortly after discharge (4.9 ± 2 days). The primary endpoint was the composite of all-cause mortality or HF-related rehospitalization at 30 days and at 1 year. All-cause mortality alone at one year was also a major endpoint. HF-related rehospitalizations alone were secondary end-points.

**Results:**

From February 2014 to November 2016, 522 consecutive patients attending the STOP-HF Clinic were included (57.1% women, age 82 ± 8.7 years, mean Barthel index 70 ± 25, mean Charlson comorbidity index 5.6 ± 2.2). The composite endpoint occurred in 8.6% patients at 30 days and in 38.5% at 1 year. In multivariable analysis, ST2 [hazard ratio (HR) 1.53; 95% CI 1.19–1.97; *p* = 0.001] was the only predictive biomarker at 30 days; at 1 year, both ST2 (HR 1.34; 95% CI 1.15–1.56; *p* < 0.001) and NT-proBNP (HR 1.19; 95% CI 1.02–1.40; *p* = 0.03) remained significant. The addition of ST2 and NT-proBNP into a clinical predictive model increased the AUC from 0.70 to 0.75 at 30 days (*p* = 0.02) and from 0.71 to 0.74 at 1 year (*p* < 0.05). For all-cause death at 1 year, ST2 (HR 1.50; 95% CI 1.26–1.80; p < 0.001), and CA125 (HR 1.41; 95% CI 1.21–1.63; p < 0.001) remained independent predictors in multivariable analysis. The addition of ST2 and CA125 into a clinical predictive model increased the AUC from 0.74 to 0.78 (p = 0.03). For HF-related hospitalizations, ST2 was the only predictive biomarker in multivariable analyses, both at 30 days and at 1 year.

**Conclusions:**

In a comorbid frail elderly population with HFpEF, ST2 outperformed NT-proBNP for predicting the risk of all-cause mortality or HF-related rehospitalization. ST2, a surrogate marker of inflammation and fibrosis, may be a better predictive marker in high-risk HFpEF.

**Electronic supplementary material:**

The online version of this article (10.1186/s12877-018-0807-2) contains supplementary material, which is available to authorized users.

## Background

Heart failure (HF) is associated with a high rate of readmission within 30 days after discharge [1, 2]. The rehospitalization rates are as high as 20–30% in the first month and increase further during the following year [3–6]. This represents a health, social, and economic burden [7–9], especially for elderly patients, as the prevalence of HF is higher in those over 80 years old [10, 11]. Elderly HF patients differ from younger patients in terms of etiology, comorbidities [12], and phenotype, and elderly patients have a higher prevalence of heart failure with preserved ejection fraction (HFpEF) [13, 14]. This clinical difference impacts short- and mid-term outcomes, and readmissions can be due to exacerbations of the underlying disease [15] as well as to other medical problems [16].

Developing risk prediction models to help in clinical decision making is a major challenge in this elderly HFpEF population [17]. Notably, identifying individuals who are at the highest risk of readmission could lay the groundwork for precision medicine to develop customized preventive measures [18]. Indeed, risk stratification may be refined by the use of biomarkers of different pathophysiologic processes that are not necessarily reflected by established clinical risk factors [19]. Predictive biomarker data in such special populations are scarce and the role of biomarkers in elderly subjects with HF is challenged by the presence of comorbidities [20].

Accordingly, our aim was to assess the value of a panel of 4 biomarkers for predicting 30-day and 1-year outcomes in a population of fragile elderly patients with comorbidities and mainly HFpEF. We analyzed the following biomarkers: N-terminal pro-brain natriuretic peptide (NT-proBNP; a marker of myocardial stretch and neurohormonal activation), interleukin-1 receptor-like 1 (ST2; a marker of inflammation and stretch and extracellular matrix remodeling), high-sensitivity troponin I (hs-TnI; a surrogate marker of myocardial injury), and cancer antigen 125 (CA125; a marker of systemic congestion in HF).

## Methods

### Study population

This prospective single-center investigation was performed as part of the **ST**ructured multidisciplinary outpatient clinic for **O**ld and frail **P**ost-discharge patients hospitalized for HF (STOP-HF-Clinic) study, which aimed to reduce readmission rates and facilitate the transition to primary care [21]. The STOP-HF-Clinic study included the most vulnerable patients with a primary hospital diagnosis of HF who were admitted for acutely decompensated HF to internal medicine and geriatric wards [22]. The interventions performed in the STOP-HF-Clinic study and at routine visits were reported previously [20]. At the first visit, a mean of 4.9 ± 2 days after discharge, the following were obtained from each patient: clinical, demographic, and treatment data; Charlson comorbidity index and Barthel functional score; and a blood sample.

### Analytical assays

#### ST2 assay

ST2 was measured using a high-sensitivity sandwich monoclonal immunoassay (Presage^®^ ST2 assay, Critical Diagnostics, San Diego, CA, USA). The hs-ST2 assay had a within-run coefficient of < 2.5% and a total coefficient of variation of 4%.

#### NT-proBNP assay

NT-proBNP was quantified using the AQT90 FLEX immunoassay (Radiometer Medical). According to the manufacturer, there is no detectable cross-reactivity with ANP, BNP, or CNP, and the within-day CV is < 10% in samples with NT-pro-BNP concentrations higher than 73 pg/mL.

#### CA125 assay

Ca125 was measured in an Architect i2000 platform (Abbott Diagnostics). The CV of the ARCHITECT CA 125 II assay has a CV < 10%. Samples with concentrations higher than 1000 U/mL were diluted 1:10 using the automated dilution protocol recommended by the manufacturer.

#### Hs-TnI assay

hs-TnI was measured using an Architect i2000 platform (Abbott Diagnostics). There was ≤0.1% cross-reactivity with skeletal troponin I and ≤ 1% cross-reactivity with cardiac troponin T and troponin C.

### Follow-up and endpoints

All patients had visits scheduled at regular intervals for 1 to 3 months, with additional visits as required in cases of decompensation or when need of treatment up-titration^20^. Clinical follow-up was subsequently performed by the patient’s general practitioner. The composite endpoints, i.e. all-cause mortality or HF-related rehospitalization at 30 days and at 1 year, were the primary endpoints. HF-related rehospitalization was also assessed as a secondary endpoint for both study periods. All-cause death at one year was analyzed as well. Death and hospital admissions were prospectively reviewed using electronic medical records (CP and MD).

### Statistical analysis

Categorical variables were expressed as percentages. Continuous variables were expressed as means (standard deviations [SDs]) or medians [25–75 percentiles] according to their distribution (normal or non-normal). Normal distribution was assessed with normal Q-Q plots. Correlation between the different studied biomarkers was performed using Pearson correlation test of log-transformed values of each biomarker. Kaplan-Meier survival curves were plotted for the biomarkers statistically independently associated with the one-year composite end-point and one-year all-cause death and the groups were compared using the Log-Rank test. Univariate Cox regression analyses were performed for all of the endpoints for the four studied biomarkers and also for relevant clinical covariates: age; sex; New York Heart Association functional class; diabetes mellitus; Charlson comorbidity index; Barthel index; and urea, creatinine, hemoglobin, and sodium levels. For HF-related re-hospitalization, a competing risk strategy using the Gray method was adopted, considering death as the competing risk. The four studied biomarkers were log-transformed and analyzed per 1 SD. Multivariable analyses were also performed using the backward step method, with age, sex, and the variables with statistical significance in the univariate analyses as covariates. Predictive “X*Beta” models were created from Cox regression analysis for the composite endpoint, both at 30 days and at 1 year. The best clinical predictive models without biomarkers were constructed, including age and sex and the variables with statistical significance in the univariate analyses. This approach was used because with such strategy we obtained the better AUC with clinical variables. The area under the curve (AUC) of these “X*Beta” models was then obtained. Next, the biomarkers with statistical significance in univariate analyses were added into the models, and new AUCs were calculated for the predictive biomarkers. AUC were compared by chi-squared test. Statistical analyses were performed with SPSS 15 (SPSS Inc., Chicago, IL, USA) and STATA V.13.0 (College Station, Texas, USA). A two-sided *p* < 0.05 was considered significant.

## Results

From February 2014 to November 2016, 522 consecutive patients at the STOP-HF Clinic (57.1% women, mean age 82 ± 8.7 years, 25% older than 88 years; mean LVEF 58.8% ±14) were included in the STOP-HF-Clinic study. Their clinical characteristics are shown in Table 1. The main etiologies were hypertension (39.7%), ischemia (28.7%), and valvular heart disease (13.2%). Diabetes, anemia, and renal failure were highly prevalent. The mean Barthel index was 70.5 ± 25.4, and the mean Charlson comorbidity index was 5.6 ± 2.2. Correlations between biomarkers were modest (Additional file 1: Table S1).

**Table 1:** Clinical characteristics of patients

	Total	End-point^a^	No end-point^a^	*p*-value
	*N* = 522	*N* = 201	*N* = 321	
Age, years	82.1 ± 8.7	84.2 ± 7.4	80.8 ± 9.2	< 0.001
Female sex	298 (57.1%)	123 (61.2%)	175 (54.5%)	0.1
Etiology				0.3
IHD	150 (28.7%)	45 (22.4%)	105 (32.7%)	
Dilated CM	9 (1.7%)	3 (1.5%)	6 (1.9%)	
Hypertensive CM	207 (39.7%)	85 (42.3%)	122 (38.0%)	
Alcoholic CM	7 (1.3%)	2 (1.0%)	5 (1.6%)	
Valvular disease	69 (13.2%)	31 (15.4%)	38 (11.8%)	
Other	80 (15.4%)	35 (17.4%)	45 (20.0%)	
LVEF^a^	55.8% ± 14	56.6% ± 14.2	55.4% ± 13.9	0.4
NYHA functional class				0.002
I	8 (1.5%)	1 (0.5%)	7 (2.2%)	
II	198 (37.9%)	63 (31.3%)	136 (42.4%)	
III	306 (58.6%)	134 (66.7%)	175 (54.5%)	
IV	6 (1.1%)	3 (1.5%)	3 (0.9%)	
Diabetes	274 (52.4%)	114 (56.7%)	160 (49.8%)	0.1
non insulin-dependent	157 (30.1%)	64 (31.8%)	93 (29.0%)	
insulin-dependent	117 (22.4%)	50 (24.9%)	67 (20.9%)	
Hypertension	468 (89.7%)	184 (91.5%)	284 (88.5%)	0.3
COPD	126 (24.1%)	54 (26.9%)	72 (22.4%)	0.3
Renal failure	411 (78.7%)	173 (86.1%)	238 (74.1%)	0.001
Anaemia	341 (65.3%)	141 (70.1%)	200 (62.3%)	0.07
Charlson comorbidity index	5.6 ± 2.2	6.1 ± 2.2	5.3 ± 2.1	< 0.001
Barthel index	70.5 ± 25.4	63.6 ± 26.5	74.7 ± 23.7	< 0.001
Core Readmission risk	26.3% ± 5.2	27.0% ± 5.1	25.8% ± 5.2	0.009
Urea, mg/dL	90.8 ± 49.4	107.2 ± 56.2	80.5 ± 41.8	< 0.001
Creatinine, mg/dL	1.4 (1.1–2.0)	1.5 (1.2–2.1)	1.8 (1.0–1.79)	< 0.001
Hemoglobin, g/dL	11.7 ± 1.6	11.5 ± 1.6	11.9 ± 1.7	0.01
Sodium, mmol/L	137.3 ± 3.8	136.9 ± 4.3	137.5 ± 3.5	0.08
NTproBNP, ng/L	2770 (1245–5843)	3900 (1695–7160)	2170 (1025–4635)	< 0.001
ST2, ng/mL	42.7 (30.9–63.8)	54.6 (36.7–79.3)	37.6 (29.1–52.3)	< 0.001
CA125^b^, U/ml	47 (22.4–101.1)	57.4 (26.7–131.4)	39.2 (20.2–90.2)	0.001
Hs-TnI^b^, ng/L	23.6 (12.2–61-2)	28.5 (14.8–67.5)	21.9 (10.4–57.8)	0.007

^a^Composite end-point all-cause death or heart failure hospitalization at one year

^b^Available in 466 patients

*CM* cardiomyopathy, *IHD* ischemic heart disease, *LVEF* left ventricular ejection fraction, *NYHA* New York Heart Association, *COPD* Chronic obstructive pulmonary disease, *CA125* Cancer antigen 125, *NTproBNP* N-terminal pro-brain natriuretic peptide; *hs-TnI* high sensitivity troponin I, *ST2* Interleukin-1 receptor-like 1

### Endpoints

HF-related rehospitalization occurred in 36 patients (6.9%) at 30 days and in 137 (26.2%) at 1 year. Death occurred in 13 patients (2.5%) at 30 days and in 121 (23.2%) at 1 year. Finally, the composite endpoint occurred in 45 patients (8.6%) at 30 days and in 201 (38.5%) at 1 year.

At 30 days, diabetes (*p* = 0.01), urea (*p* < 0.05), Barthel index (*p* = 0.008), Charlson comorbidity index (*p* < 0.001), NT-proBNP (*p* = 0.001), and ST2 (*p* < 0.001) were all associated with the primary composite endpoint in the univariate analyses (Table 2). Diabetes (*p* = 0.007), Charlson comorbidity index (*p* = 0.001), NT-proBNP (*p* = 0.04), and ST2 (*p* < 0.001) were associated with HF-related rehospitalization (Additional file 2: Table S2). In multivariable analysis, female sex (*p* = 0.01), Charlson comorbidity index (p < 0.001), and ST2 (p = 0.001) were the only independent variables associated with the primary composite endpoint (Table 2) and also with HF-related rehospitalization [female sex (p = 0.04), Charlson comorbidity index (*p* = 0.003), and ST2 (*p* = 0.008)] (Additional file 2: Table S2).

**Table 2:** Cox regression analyses for the 30-day primary endpoint (all-cause death or HF-related rehospitalization)

	30-day composite endpoint					
	Univariate analysis			Multivariate analysis		
	HR	95%CI	*p*-value	HR	95%CI	*p*-value
Age	1.03	0.99–1.07	0.2	–	–	–
Female sex	1.54	0.83–2.87	0.2	2.28	1.19–4.37	0.01
NYHA	1.08	0.63–1.87	0.8			
Diabetes	1.59	1.11–2.56	0.01	–	–	–
Charlson comorbidity index	1.28	1.14–1.44	< 0.001	1.26	1.11–1.43	< 0.001
Barthel index	0.99	0.98–1.00	0.008	–	–	–
Urea	1.01	1.00–1.01	< 0.05	–	–	–
Creatinine	0.99	0.93–1.06	0.7			
Hb	0.93	0.77–1.11	0.4			
Na	1.05	0.97–1.14	0.2			
NT-proBNP^a^	1.63	1.21–2.19	0.001	–	–	–
ST2^a^	1.67	1.33–2.11	< 0.001	1.53	1.19–1.97	0.001
CA125^a^	1.12	0.83–1.51	0.5			
Hs-TnI^a^	1.23	0.92–1.63	0.2			

^a^Log-transformed and per 1 SD

*CA125* cancer antigen 125, *NT-proBNP* N-terminal pro-brain natriuretic peptide, *hs-TnI* high-sensitivity troponin I, *ST2* Interleukin-1 receptor-like 1

**Fig. 1:**
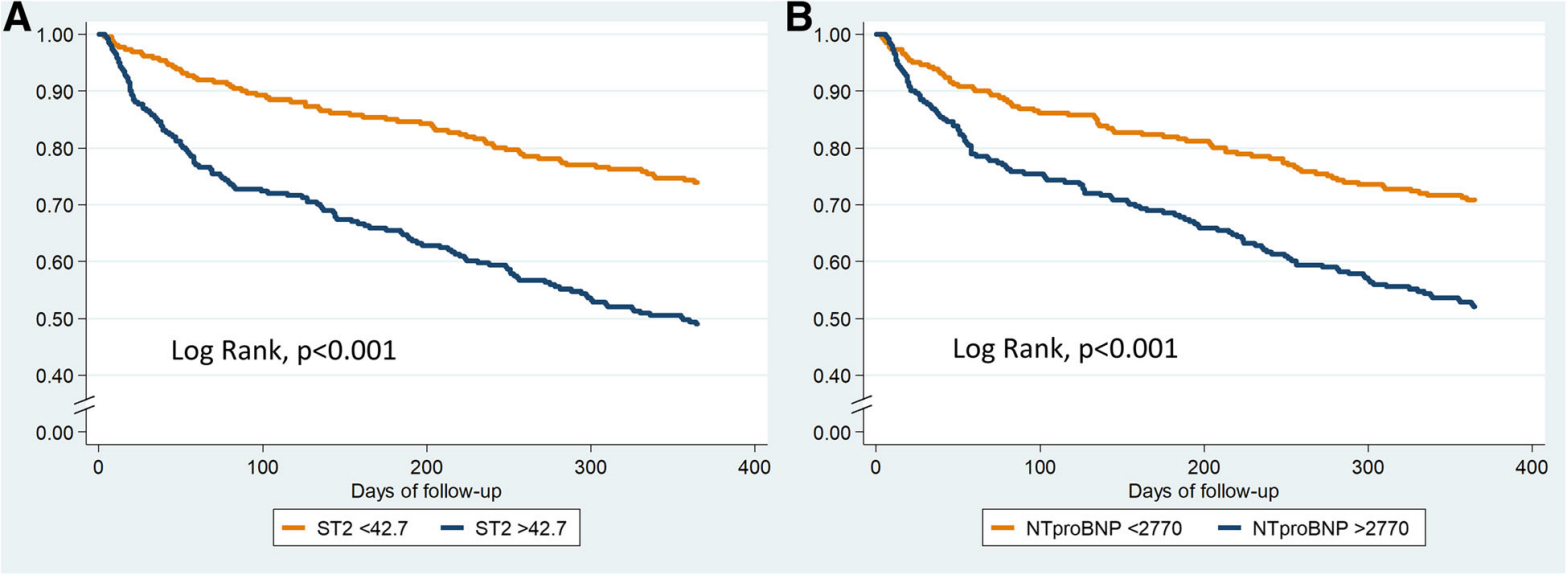
Kaplan-Meier event-free survival curves for the composite end-point relative to serum levels of ST2 (**a**) and NTproBNP (**b**) above or below the median

**Fig. 2:**
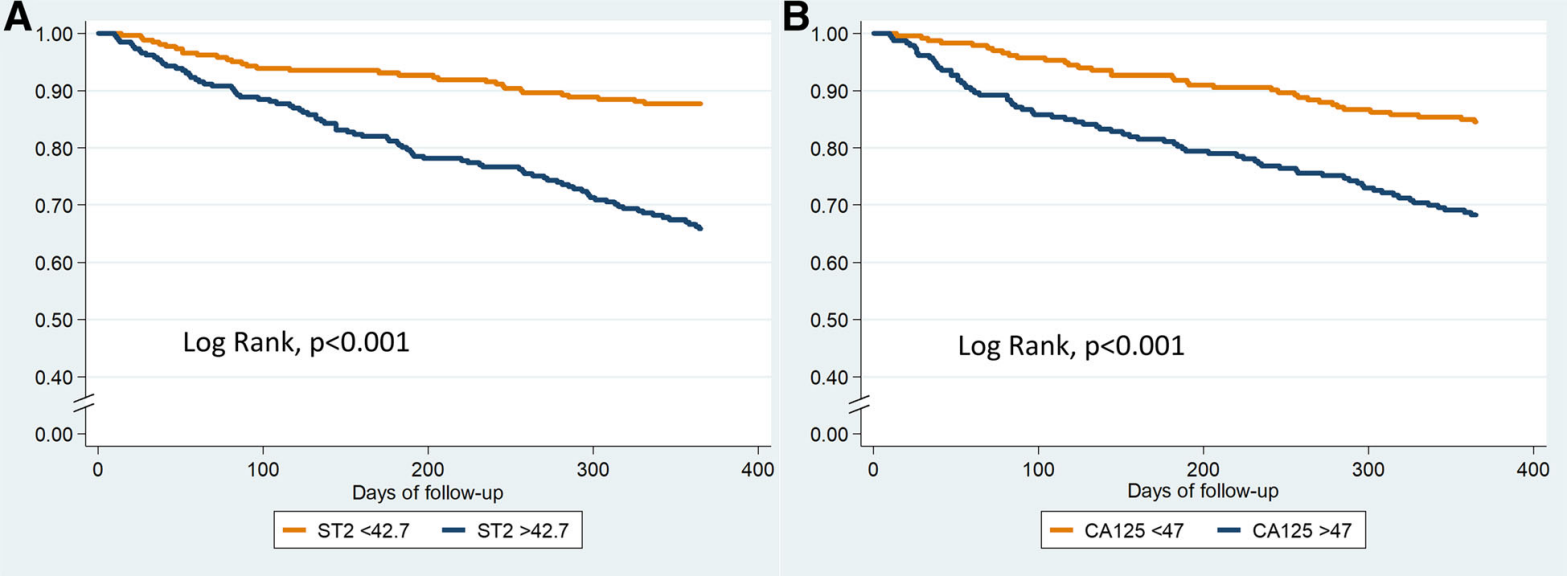
Kaplan-Meier survival curves for all-cause death relative to serum levels of ST2 (**a**) and CA125 (**b**) above or below the median

Table 3 and Additional file 3: Table S3 show the results of the univariable and multivariable analyses for the composite endpoint and HF-related rehospitalization at one year, respectively. In multivariate analysis, age (p = 0.003), female sex (*p* = 0.002), urea (*p* < 0.001), Charlson comorbidity index (p = 0.008), NT-proBNP (p = 0.04), and ST2 (p < 0.001) remained significantly associated with the composite endpoint (Table 3); only female sex (p = 0.01), urea (p < 0.001), and ST2 (*p* = 0.001) remained significantly associated with HF-related rehospitalization (Additional file 3: Table S3). Figure 1 shows Kaplan-Meier event-free survival curves for the composite end-point relative to circulating levels of ST2 and NTproBNP. Table 4 shows Cox regression analysis results for all-cause death at 1 year. Age (p < 0.001), female sex (p = 0.008), Charlson comorbidity index (p = 0.001), Barthel index (p = 0.008), ST2 (p < 0.001), and CA125 (p = 0.003) remained independent predictors for all-cause death at 1 year. Figure 2 shows Kaplan-Meier survival curves for all-cause death relative to circulating levels of ST2 and CA125.

**Table 3:** Cox regression analyses for the 1-year composite endpoint (all-cause death or HF related rehospitalization)

	1-year composite endpoint					
	Univariate analysis			Multivariate analysis		
	HR	95%CI	p-value	HR	95%CI	p-value
Age	1.04	1.02–1.06	< 0.001	1.03	1.01–1.06	0.003
Female sex	1.28	0.96–1.70	0.09	1.64	1.20–2.23	0.002
NYHA	1.54	1.18–2.01	0.002	–	–	–
Diabetes	1.16	0.98–1.38	0.09			
Charlson comorbidity index	1.15	1.08–1.22	< 0.001	1.10	1.03–1.18	0.008
Barthel index	0.99	0.98–0.99	< 0.001	–	–	–
Urea	1.01	1.01–1.01	< 0.001	1.01	1.00–1.01	< 0.001
Creatinine	1.00	1.00–1.01	0.3	–	–	–
Hb	0.90	0.82–0.98	0.01	–	–	–
Na	0.97	0.93–1.00	0.07			
NT-proBNP^a^	1.43	1.25–1.65	< 0.001	1.18	1.00–1.38	0.04
ST2^a^	1.63	1.44–1.85	< 0.001	1.41	1.21–1.63	< 0.001
CA125^a^	1.32	1.15–1.52	< 0.001	–	–	–
Hs-TnI^a^	1.21	1.05–1.39	0.007	–	–	–

^a^Log-transformed and per 1 SD

*CA125* cancer antigen 125, *NT-proBNP* N-terminal pro-brain natriuretic peptide, *hs-TnI* high-sensitivity troponin I, *ST2* Interleukin-1 receptor-like 1

**Table 4:** Cox regression analyses for 1-year all-cause death

	1-year all-cause death					
	Univariate			Multivariate		
	HR	95%CI	*p*-value	HR	95%CI	*p*-value
Age	1.08	1.05–1.11	< 0.001	1.08	1.05–1.12	< 0.001
Female Sex	1.28	0.89–1.85	0.2	1.73	1.16–2.59	0.008
NYHA	2.01	1.40–2.90	< 0.001	–	–	–
Diabetes	1.03	0.83–1.29	0.8			
Charlson comorbidity index	1.15	1.05–1.26	0.002	1.17	1.06–1.28	0.001
Barthel index	0.98	0.97–0.99	< 0.001	0.99	0.98–1.00	0.008
Urea	1.01	1.00–1.01	< 0.001	–	–	–
Creatinine	1.00	1.00–1.01	0.4			
Hb	0.87	0.77–0.97	0.02	–	–	–
Na	0.97	0.93–1.02	0.2			
NT-proBNP^a^	1.65	1.37–1.98	< 0.001	–	–	–
ST2^a^	1.86	1.59–2.18	< 0.001	1.45	1.21–1.74	< 0.001
CA125^a^	1.58	1.33–1.89	< 0.001	1.35	1.11–1.65	0.003
Hs-TnI^a^	1.29	1.09–1.52	0.003	–	–	–

^a^Log-transformed and per 1 SD

*CA125* cancer antigen 125, *NT-proBNP* N-terminal pro-brain natriuretic peptide, *hs-TnI* high-sensitivity troponin I, *ST2* Interleukin-1 receptor-like 1

### Risk prediction

**Fig. 3:**
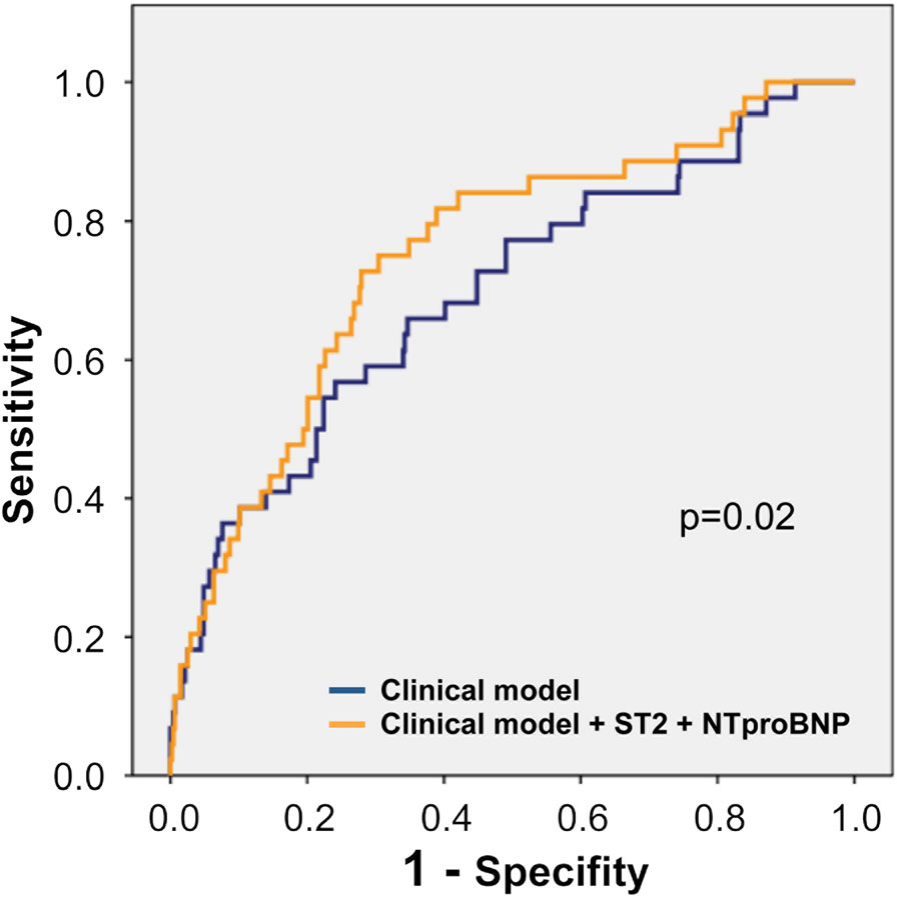
The area under the curve (AUC) for the primary composite endpoint at 30 days for the clinical model and the model with ST2 and NT-proBNP. The clinical model included age, sex, diabetes, urea, Charlson comorbidity index, and Barthel index

Risk prediction of the composite endpoint at 30 days for the clinical model, including age, sex, diabetes, urea, Charlson comorbidity index, and Barthel index, revealed an AUC of 0.696 [0.609–0.783]. The addition of NT-proBNP and ST2 to the clinical model increased the AUC to 0.750 [0.673–0.827] (*p* = 0.02) (Fig. 3). Further, reclassification significantly improved [NRI 0.40 (0.08–0.80), IDI 0.02 (− 0.002–0.06)].

Risk prediction of the composite endpoint at 1 year for the clinical model that included age, sex, NYHA class, urea, hemoglobin, Charlson comorbidity index, and Barthel index revealed an AUC of 0.711 [0.665–0.757]. Addition of the 4 studied biomarkers increased the AUC to 0.736 [0.689–0.783] (*p* = 0.05), and provided significantly better patient reclassification [NRI 0.40 (0.21–0.58), IDI 0.04 (0.01–0.08)]. However, if only NT-proBNP and ST2 (the two biomarkers that were statistically significant in the multivariable analysis) were added to the clinical model, the AUC was virtually the same (0.735 [0.690–0.780]) (*p* < 0.05 relative to clinical model), and reclassification improvement remained significant as well [NRI 0.38 (0.19–0.55), IDI 0.04 (0.01–0.07)].

Risk prediction for all-cause death at 1 year for the clinical model that included the same covariables revealed an AUC of 0.746 [0.697–0.795]. Addition of the 4 studied biomarkers increased the AUC to 0.789 [0.739–0.838] (*p = 0.01*). Again, reclassification significantly improved [NRI 0.56 (0.36–0.78), IDI 0.06 (0.02–0.12]. However, if only ST2 and CA125 (the two biomarkers that were statistically significant in the multivariable analysis) were added to the clinical model, the AUC was practically the same (0.786 [0.736–0.836]) (*p = 0.02* relative to clinical model), as well as reclassification [NRI 0.60 (0.36–0.80), IDI 0.06 (0.02–0.11)].

## Discussion

This study comprehensively analyzed clinical variables and predictive biomarkers to determine their association with 30-day and 1-year adverse events in comorbid frail elderly patients with mainly HFpEF. In terms of clinical prediction, higher multimorbidity is associated with higher risk of all-cause death and readmission both at 30 days [16, 23] and at 1 year [24] despite high-quality multidisciplinary management, especially in the elderly [24, 25]. In our cohort, the Charlson Index as a measure of comorbidity was associated with both composite endpoints at 30 days and 1 year and also with HF-related rehospitalization at 30 days. The prognostic role of the Charlson comorbidity index has been described previously [5, 26], but in our study, it remained independently associated with outcome despite the inclusion of strong predictive biomarker variables. Remarkably, both the primary composite endpoint and the secondary endpoint of 30-day HF rehospitalization were only associated with female sex, Charlson comorbidity index, and ST2.

Notably, predictive biomarkers showed value beyond that of clinical risk factors, since circulating biomarkers have the potential to predict the risk of recurrent hospitalization or death [27, 28]. In our elderly cohort, ST2 emerged as the strongest predictor in both univariate and multivariate analysis for all of the endpoints, which is in line with recent reports [29]. In our study, NT-proBNP, hs-TnI, and CA-125 lost their statistical significance when ST2 and clinical variables were included in the model.

The addiction of circulating ST2 to our model was based on its value as a surrogate of inflammation, stretch, and extracellular matrix status as well as on the observation that the ST2 concentration is not affected by age, renal function [30], or body mass index [31]. These characteristics are highly desirable in a marker that will be used in an elderly population with much comorbidity. Our current data is in line with the notion that ST2 reflects systemic inflammatory disease involving multiple organ systems [32] and that it is related to HFpEF development and to the proinflammatory and profibrotic state [33]. Indeed, it was recently postulated that ST2 is the new “gold standard” for HF prognostication and monitoring [29].

NT-proBNP, a marker of myocardial stretch and neurohormonal activation, is the only biomarker that is currently included in HF guidelines [34] and it has shown incremental diagnostic and prognostic value in HFrEF and HFpEF [35, 36]. However, certain conditions, which are highly prevalent in our patient population, can make it difficult to interpret this versatile biomarker. These conditions include the presence of underlying structural cardiopulmonary diseases (chronic obstructive disease, pulmonary hypertension), anemia, advanced age, atrial fibrillation, female sex, and renal failure, all of which result in high levels of NT-proBNP. By contrast, obesity is well recognized as a condition that reduces circulating NT-proBNP levels. In the current study, NT-proBNP lost its discriminative value: it showed a significant association with the composite endpoint at 1 year, but no significant association with HF-related rehospitalization or 30-day outcomes.

Hs-TnI was included in our analysis because it is generally accepted that troponins offer valuable information for risk stratification during acute decompensation in patients with HFpEF [37]. High levels of hs-TnI have been associated with worse in-hospital, 30-day, and 1-year survival and with longer length of stay and more readmission risk within 30 days [37]. Nevertheless, our data showed that when a multi-biomarker panel was included in the multivariable analysis, hs-TnI lost its predictive value in this high-risk population. Further, as is the case for natriuretic peptides, troponins are strongly influenced by age, history of diabetes mellitus, and lower estimated glomerular filtration rate [38].

CA125 is an interesting biomarker. It has traditionally been linked to ovarian neoplasm, but more recently it has been associated with tissue congestion. Núñez et al. [39] found that CA125 as measured during acute HF hospitalization is an independent predictor of mortality [39]. In our cohort, CA125 also emerged as an independent prognosticator of all-cause death at 1 year, but it did not provide additional value to the primary endpoint at the 30-day or 1-year follow-up. In the STOP-HF Clinic [20] special attention has been paid to treatment of heart congestion; indeed, 100% of the patients were on diuretic therapy, and more than 300 infusions of IV furosemide were administered during the first month of follow-up. The neutral effect of CA125 regarding the composite endpoints may partly be explained by the fact that its assessment was not performed during the acute phase of HF decompensation, where prior works pointed out its value as marker of fluid overload. However, it is remarkable that CA125 remained independently associated with 1-year all-cause mortality regardless it was not measured during the maximum wet phase. Further studies are needed to better understand the value of CA125 in HF.

Historically, it has been much more difficult to estimate the risk of HF hospitalization than to estimate the risk of death [40]. The high rate of readmissions within 30 days post-discharge and during the following year in ageing and comorbid patients highlights the urgency of establishing predictive models with acceptable performance in such patients to guide clinical management and decision making. To our knowledge, the predictive models based on biomarkers that were developed in this study are the first that can be used in this comorbid fragile elderly population with HF. The BIOSTAT-CHF study recently developed and validated three risk models that included biomarkers (natriuretic peptides) to predict all-cause mortality, HF-related hospitalization, and the composite endpoint in a cohort of patients with worsening HF. The patients included in their cohort were a mixture of in-hospital and ambulatory patients with a mean age of 69 years, 27% female, and mean LVEF of 31% [41]; in other words, theirs was a relatively young and mainly male HFrEF population that was quite different from our study cohort. These researchers obtained C-statistic values of 0.73, 0.69, and 0.71 for all-cause mortality, HF-related hospitalization, and the composite endpoint, respectively; these were lower than those reported in the present study, and their follow-up time was 21 months. No short-term predictions were performed.

### Limitations

Our study was an observational single-center study with a cohort of elderly patients with comorbidities that mainly had HF of hypertensive etiology and preserved ejection fraction. As such, our results cannot be extrapolated to a global HF population. In fact, the absence of predictive models in such patients may be a strength rather than a limitation given the growing number of HF patients with this phenotype.

The biomarker selection was based on previously described predictive value of the biomarkers, on other information about HF pathways, and on its availability for use in routine clinical practice. The optimal panel of biomarkers remains debatable. One major limitation of this study is the absence of a validation cohort. However, we have not been able to identify a post-discharge cohort with similar clinical characteristics with available multi-biomarker data.

## Conclusions

In a comorbid fragile elderly population with mainly HFpEF, ST2 outperformed NT-proBNP for risk prediction of the composite primary endpoint (all-cause mortality or HF-related rehospitalization). Regarding 1-year all-cause mortality, ST2 together with CA125 were independently associated with higher risk and the addition of both biomarkers improved the discriminative accuracy of clinical data. ST2 is a three-in-one biomarker as a surrogate of inflammation, stretch, and extracellular matrix remodeling, and it may be the preferred biomarker for short- and long-term prediction in a high-risk HF population like ours. Comorbidities, as represented by the Charlson comorbidity index, also strongly impacted these patients’ outcomes. Further studies in similar populations are needed to confirm our results.

## Additional files

Additional file 1: **Table S1.** Correlations between studied biomarkers. Correlation between the different studied biomarkers was performed using Pearson correlation test of log-transformed values of each biomarker. (DOCX 25 kb)

Additional file 2: **Table S2.** Cox regression analyses for 30-day rehospitalization. A competing risk strategy using the Gray method was adopted, considering death as the competing risk in both univariate and multivariate Cox regression analyses. (DOCX 27 kb)

Additional file 3: **Table S3.** Cox regression analyses for 1-year HF-related hospitalization. A competing risk strategy using the Gray method was adopted, considering death as the competing risk in both univariate and multivariate Cox regression analyses. (DOCX 27 kb)

## Abbreviations

CA125: Cancer antigen 125
HF: Heart failure
HFpEF: Heart failure with preserved ejection fraction
Hs-TnI: High-sensitivity troponin I
NT-proBNP: N-terminal pro-brain natriuretic peptide
ST2: Interleukin-1 receptor-like 1
STOP-HF-Clinic: **ST**ructured multidisciplinary outpatient clinic for **O**ld and frail **P**ost-discharge patients hospitalized for HF
