# Supplementary material for: Predictive biomarkers for death and rehospitalization in comorbid frail elderly heart failure patients
Source: BMC Geriatr. 2018 May 9;18:109. doi: 10.1186/s12877-018-0807-2 (PMC5944009; doi:10.1186/s12877-018-0807-2)
Supplement: Supplementary file 1 — Table S1. Correlations between studied biomarkers. Correlation between the different studied biomarkers was performed using Pearson correlation test of log-transformed values of each biomarker. (DOCX 25 kb) [file 12877_2018_807_MOESM1_ESM.docx]

**Supplementary table S1. Correlations between studied biomarkers**

|  | NTproBNP | Hs-TnI | CA 125 |
| --- | --- | --- | --- |
| ST2 | R=0.32, p<0.001 | R=0.23, p<0.001 | R=0.32, p<0.001 |
| NTproBNP |  | R=0.35, p<0.001 | R=0.34, p<0.001 |
| Hs-TnI |  |  | R=0.18, p<0.001 |

R=Pearson correlation between log-transformed form of biomarker

CA125 = cancer antigen 125, NT-proBNP = N-terminal pro-brain natriuretic peptide, hs-TnI = high-sensitivity troponin I, ST2 = Interleukin-1 receptor-like 1.
