# Supplementary material for: Predictive biomarkers for death and rehospitalization in comorbid frail elderly heart failure patients
Source: BMC Geriatr. 2018 May 9;18:109. doi: 10.1186/s12877-018-0807-2 (PMC5944009; doi:10.1186/s12877-018-0807-2)
Supplement: Supplementary file 2 — Table S2. Cox regression analyses for 30-day rehospitalization. A competing risk strategy using the Gray method was adopted, considering death as the competing risk in both univariate and multivariate Cox regression analyses. (DOCX 27 kb) [file 12877_2018_807_MOESM2_ESM.docx]

**Supplementary Table S2.** **Cox regression analysis for 30-day rehospitalization.**

|  | **30-day HF-related rehospitalization^#^** | | | | | |
| --- | --- | --- | --- | --- | --- | --- |
|  | **Univariate analysis** | | | **Multivariate analysis** | | |
|  | **HR** | **95%CI** | **p-value** | **HR** | **95%CI** | **p-value** |
| Age | 1.04 | 0.98-1.05 | 0.4 | -- | --- | -- |
| Female sex | 1.54 | 0.77-3.06 | 0.2 | 2.07 | 1.02-4.22 | 0.04 |
| NYHA | 0.79 | 0.44-1.41 | 0.4 |  |  |  |
| Diabetes | 1.75 | 1.16-2.63 | 0.007 | -- | --- | -- |
| Charlson comorbidity index | 1.26 | 1.09-1.45 | 0.001 | 1.26 | 1.08-1.46 | 0.003 |
| Barthel index | 0.99 | 0.98-1.00 | 0.2 |  |  |  |
| Urea | 1.01 | 1.00-1.01 | 0.08 |  |  |  |
| Creatinine | 0.99 | 0.98-1.0 | 0.2 |  |  |  |
| Hb | 0.87 | 0.72-1.06 | 0.2 |  |  |  |
| Na | 1.04 | 0.95-1.15 | 0.4 |  |  |  |
| NT-proBNP* | 1.37 | 1.01-1.86 | 0.04 | -- | --- | -- |
| ST2* | 1.53 | 1.21-1.92 | <0.001 | 1.39 | 1.09-1.78 | 0.008 |
| CA125* | 1.01 | 0.72-1.41 | 1.0 |  |  |  |
| Hs-TnI* | 1.11 | 0.76-1.62 | 0.6 |  |  |  |

*Log-transformed and per 1 SD

**^#^**Death has been taken into account as competitive risk.

CA125 = cancer antigen 125, NT-proBNP = N-terminal pro-brain natriuretic peptide, hs-TnI = high-sensitivity troponin I, ST2 = Interleukin-1 receptor-like 1.
