# Supplementary material for: Predictive biomarkers for death and rehospitalization in comorbid frail elderly heart failure patients
Source: BMC Geriatr. 2018 May 9;18:109. doi: 10.1186/s12877-018-0807-2 (PMC5944009; doi:10.1186/s12877-018-0807-2)
Supplement: Supplementary file 3 — Table S3. Cox regression analyses for 1-year HF-related hospitalization. A competing risk strategy using the Gray method was adopted, considering death as the competing risk in both univariate and multivariate Cox regression analyses. (DOCX 27 kb) [file 12877_2018_807_MOESM3_ESM.docx]

**Supplementary Table S3.** **Cox regression analysis for 1-year HF-related hospitalization.**

|  | **1-year HF-hospitalization^#^** | | | | | |
| --- | --- | --- | --- | --- | --- | --- |
|  | **Univariate** | | | **Multivariate** | | |
|  | **HR** | **95%CI** | **p-value** | **HR** | **95%CI** | **p-value** |
| Age | 1.02 | 1.00-1.04 | <0.05 | -- | --- | -- |
| Female sex | 1.56 | 1.10-2.21 | 0.01 | 1.65 | 1.14-2.40 | 0.01 |
| NYHA | 1.40 | 1.02-1.93 | 0.04 | -- | --- | -- |
| Diabetes | 1.27 | 1.03-1.57 | 0.03 | -- | --- | -- |
| Charlson comorbidity index | 1.12 | 1.05-1.21 | 0.001 | -- | --- | -- |
| Barthel index | 0.99 | 0.98-0.99 | <0.001 | -- | --- | -- |
| Urea | 1.01 | 1.01-1.01 | <0.001 | 1.01 | 1.00-1.01 | <0.001 |
| Creatinine | 1.00 | 0.99-1.01 | 0.7 |  |  |  |
| Hb | 0.90 | 0.81-1.00 | <0.05 | -- | --- | -- |
| Na | 0.97 | 0.92-1.01 | 0.2 |  |  |  |
| NT-proBNP* | 1.30 | 1.10-1.53 | 0.002 | -- | --- | -- |
| ST2* | 1.42 | 1.22-1.65 | <0.001 | 1.31 | 1.11-1.55 | 0.001 |
| CA125* | 1.23 | 1.04-1.47 | 0.02 | -- | --- | -- |
| Hs-TnI* | 1.10 | 0.93-1.30 | 0.3 |  |  |  |

*Log-transformed and per 1 SD

**^#^**Death has been taken into account as competitive risk.

CA125 = cancer antigen 125, NT-proBNP = N-terminal pro-brain natriuretic peptide, hs-TnI = high-sensitivity troponin I, ST2 = Interleukin-1 receptor-like 1.
